# A Seasonal Time-Series Model Based on Gene Expression Programming for Predicting Financial Distress

**DOI:** 10.1155/2018/1067350

**Published:** 2018-03-22

**Authors:** Ching-Hsue Cheng, Chia-Pang Chan, Jun-He Yang

**Affiliations:** Department of Information Management, National Yunlin University of Science and Technology, 123 University Road, Section 3, Douliou, Yunlin 64002, Taiwan

## Abstract

The issue of financial distress prediction plays an important and challenging research topic in the financial field. Currently, there have been many methods for predicting firm bankruptcy and financial crisis, including the artificial intelligence and the traditional statistical methods, and the past studies have shown that the prediction result of the artificial intelligence method is better than the traditional statistical method. Financial statements are quarterly reports; hence, the financial crisis of companies is seasonal time-series data, and the attribute data affecting the financial distress of companies is nonlinear and nonstationary time-series data with fluctuations. Therefore, this study employed the nonlinear attribute selection method to build a nonlinear financial distress prediction model: that is, this paper proposed a novel seasonal time-series gene expression programming model for predicting the financial distress of companies. The proposed model has several advantages including the following: (i) the proposed model is different from the previous models lacking the concept of time series; (ii) the proposed integrated attribute selection method can find the core attributes and reduce high dimensional data; and (iii) the proposed model can generate the rules and mathematical formulas of financial distress for providing references to the investors and decision makers. The result shows that the proposed method is better than the listing classifiers under three criteria; hence, the proposed model has competitive advantages in predicting the financial distress of companies.

## 1. Introduction

Financial distress is also known as “financial crisis,” referring to the situation when cash flow is not sufficient to compensate the current debt. Listed companies having financial crisis or bankruptcy may affect the stability of the entire capital market or may even cause panic of investors and economic losses, such that damages on the interest parties of the shareholders, creditors, investors, and company employees could be serious. If there is a feasible financial crisis warning model for listed companies, during the early stage of the occurrence of financial crisis, to business manager, the countermeasures can be adopted early to prevent the expansion of damages. To investors, the financial crisis warning model can also strengthen the capital market in order to provide guarantees to investors whom may not be aware of the operation status of the companies. Therefore, a feasible warning model is able to detect problems of a listed company early in order to prevent significant losses of investors.

Currently, there are a great number of financial crisis prediction models, and each model has its own applicable timing, advantages, and disadvantages. The financial crisis prediction models can be divided into the statistical method and the artificial intelligence method. The statistical method requires the data variables to satisfy certain assumptions, whereas the artificial intelligence method requires no assumption of any probability distribution. In addition, many artificial intelligence methods refer to the free-model method [1], which only requires adjustments of some environmental parameters for the machine learning to train the output of the prediction rules. From the literature, this study finds that early researches have some drawbacks:

1. The selected index in the shortcoming of some of previous researches is based on personal experience and opinion.
2. The variables of the statistical model need to follow relevant assumptions [2].
3. Most of the past researches use the linear attribute selection method or no attribute selection to build prediction model.
4. The traditional GA uses the fixed length coding method that performance is poor while facing complicated problems. However, GP uses nonlinear and dynamic data structure for coding, which is capable of handling complicated problem [3].

For properly handling the above problems, this paper proposed a novel seasonal time-series gene expression programming model for predicting the financial distress of companies. The proposed model has some foci including the following: (1) the proposed model is seasonal time-series model; (2) the proposed model can find the core attributes and reduce high dimensional data; (3) the proposed model can generate the decision rules and mathematical formulas to build a feasible warning model to be provided to the investors and decision makers as references.

The remainder of this paper is organized as follows.  Section 2 provides the related literature review, including financial crisis, attribute selection methods, related data mining techniques, and gene expression programming.  Section 3 describes the proposed method and algorithm. The experimental results and comparison are presented in Section 4. The last section is the conclusion.

## 2. Literature Review

This section introduced the related works including financial crisis, attribute selection methods, related data mining techniques, and gene expression programming in the following.

### 2.1. Financial Crisis

In general, financial crisis referred to a company having insufficient cash flow to pay for its debt. Beaver [4] defined the financial distress as bankruptcy, preferred dividends in arrears, bank overdrafts, and irredeemable debenture. Deakin [5] believed that financial distress included companies that had bankrupted, without ability to pay for debt or had undergone liquidation for creditors' benefits. Carmichael [6] proposed the opinion that financial distress referred to hindrance to businesses performed by corporates, and the actual demonstrations thereof included insufficient liquidity, insufficient rights, insufficient funds, and delinquencies. Foster [7] expressed that the so-called financial distress referred to a company having serious asset for cash problem, and the resolution of such problem requiring the reliance on the transformation of the operation method or existence format of the company. Morris [8] listed out 12 signs indicating a corporate in its financial distress in a descending order of severity. Quinlan [9] pointed out that financial crisis referred to the situation where the operating cash flow of a company was insufficient to satisfy the current condition of debt such that the company was forced to adopt corrective actions. The definition of bankruptcy is a legal outcome in which a court discovers that the corporate has no ability to pay for debt or the action of expropriation is performed after pursuing the payment of debt. Altman [10] defined that the finical crisis referred to the restructuring for the bankruptcy determined legally. Ohlson [11] also defined a company in financial crisis as the one satisfying the standard for determination of bankruptcy legally. The definition of financial crisis has become clearer and it includes the meaning of early prevention. If it can be more definite, it would allow companies to discover and prevent in advance such that there is also a great chance for saving such companies. In other words, financial crisis is not equivalent to bankruptcy. On the contrary, a company that bankrupts must have experienced financial crisis.

Sun et al. [12] proposed a definition of financial crisis and showed various definitions of different viewpoints for financial crisis. Different scholars may have different interpretations according to the objectives of their researches. From most of the definitions, they can be mainly divided into two types. (1) In theoretical analysis, financial crisis has different degrees, in which a mild financial crisis may involve temporary difficulties in cash flow, and a serious financial crisis may involve corporate failure or bankruptcy. When a corporate is in a financial crisis, it may experience a dynamic change between these two extreme ends. (2) In empirical research, they proposed clearly the criteria of research sampling and the restrictions of data availability. And financial crisis is often clearly defined to be some situations, and such situations clearly describe financial difficulty such as statutory bankruptcy.

For financial crisis, the related methods included the traditional statistical method and the artificial intelligence method. However, the variable of statistical model requires some assumptions [2]: (a) variables have normal distributions, (b) the variables need to be independent of each other, (c) variables must have a high discriminative ability of separating solvent companies from insolvent ones, (d) in dataset, each record must be complete, and (e) corporate classification shall be clearly defined, meaning that companies belonging to a certain class shall not belong to another class. In contrast to the statistical models, artificial intelligence has the parallel computation ability such that it is able to handle nonlinear system problems, and artificial intelligence is widely used in the field of financial crisis prediction. Artificial intelligence methods include the neural network (NN), genetic algorithm (GA), rough set theory (RST), case-based reasoning (CBR), support vector machine (SVM), and *k*-nearest neighbor (KNN) methods.

Nevertheless, statistical methods and artificial intelligence methods have their own merits and drawbacks. For example, the widely used statistical multiple discriminant analysis (MDA) has simple and outstanding interpretation advantage; however, strict statistic assumption limits its application, and it is a static determination model. In contrast to the back-propagation neutral network of artificial intelligence, its application requires no assumption of any probability distribution, and the back-propagation neural network is an effective tool for nonlinear system model. Therefore, many researchers use three hidden layer back-propagation neural networks to predict financial crisis.

Many researches used the financial ratio to build financial crisis prediction model; this paper summarized related studies for data sample and results as Table 1. From Table 1, we can find that in recent years, many researches utilized the attribute selection and classifier of artificial intelligent to build financial crisis models, and these methods primarily are based on the practical industrial data in order to develop the financial crisis prediction model.  Table 2 is a list of financial ratio used in the financial crisis prediction model.  Table 2 shows that the researches with the use of financial ratio have gradually increased.

### 2.2. Attribute Selection

Attribute selections include (1) subjective attribute selection: the researcher asks experts to select attributes based on consensus decision, such as Delphi panel and analytic hierarchy process (AHP); (2) objective attribute selection: since 1970, the objective attribute selection has been widely used in various research fields, such as the statistical model analysis [13], machine learning [14], and data mining [15]. Attribute selection is an important step in the data mining preprocessing, and its primary purpose is to select important and useful information from vast amount of information, referring to selection of useful attributes and deletion of unnecessary and irrelevant attributes. Through the step of attribute selection, a more effective outcome can be achieved [16].

The algorithms for attribute selection are based on different rules of evaluation, which can be generally divided into two types: one comprising two methods of filter and wrapper, and the other is known as the embedded method. The filter method is performed for the attribute evaluation based on the characteristic of data rather than using particular algorithm to perform the attribute selection, such as the use of distance, correlation, or consistency for evaluation. Since filter method does not require the use of any algorithms, it has a relatively faster computation speed. The wrapper method uses particular algorithm to perform attribute selection; therefore, it often demonstrates relatively better performance; however, it requires longer computation time and higher costs. The embedded method (hybrid method) integrates the filter and wrapper methods by using different bases and computation processes to perform the action of attribute selection [16]. Next the following introduced the attribute selection methods used in this research.

#### 2.2.1. Decision Tree (DT)

Since 1960, many scholars have started to use tree structures to perform data analysis, including AID, ID3, CHAID, and FACT, which shows that decision tree is a widely used classification prediction tool. The decision tree is a tree structure similar to a flowchart, in which each node represents a test on the attribute, each branch represents an output of a completed test, and the leaf nodes represent the classification or distribution of the classification [26]. ID3 [27] is a decision tree algorithm based on the decision of information theory, and its basic strategy is to pick attribute with high information gain. C4.5 is obtained based on the expansion of ID3, which has been designed by Quinlan [9] and used for solving some issues that cannot be handled by ID3 [28, 29], such as reducing the errors of tree pruned, processing continuous attribute, preventing overfitting of data, depth of development of decision tree, determination of tree pruned, deciding an appropriate attribute selection measure, processing training data with missing value, increasing computation efficiency, and different costs to process attribute.

#### 2.2.2. Support Vector Machine (SVM)

Support vector machine is proposed by Cortes and Vapnik [30], which was a supervised learning method and widely used in classification and regression analysis. SVM is a concept for performing classification on hyperplane, which can be one or a multiple of hyperplanes and seeks to find a hyperplane among these hyperplanes in order to maximize the margin between two classes. A nonlinear classifier is to apply kernel function on SVM, and the formality of the computation result is similar to the linear type, except that each inner product is replaced by the kernel function. Therefore, it allows the algorithm to be applied to converting the attribute space to the hyperplane with maximized margin, which can also be converted to nonlinear high dimensional space. Consequently, classifier is on the hyperplane of high dimensional attribute space. The advantages of SVM [31] include the following: (a) nonlinear determination classifier allows the theoretical and actual results to match greatly; (b) the problem of overfitting rarely occurs; (c) the problem of overly large dimension due to excessive characteristics is not obvious; (d) it does not converge at local optimal solution but converges at the overall optimal solution; and (e) flexibly application of kernel function.

#### 2.2.3. Multilayer Perceptron (MLP)

In 1950, scientists mimicked the operation method of human brain and started to propose the “Perception” neural element model, which was known as the artificial neural network (ANN). In 1980, Hopfield neural network was proposed, and artificial neural network theory gradually drew more attention. Up to the present day, many new structures and theories are still continuously proposed, among which the most popular theory is the multilayer perception [32], which is also known as the back-propagation neural network. The structure of the back-propagation neural network includes the input layer, hidden layer, and output layer. The first layer of input layer is used for receiving external information, and the last layer of output layer is able to generate a solution model. In addition, in the input layer and the output layer, there may be one or a multiple of hidden layers, and such hidden layers are used for identifying some complicated models in the data. The advantages of MLP rely on that it is able to generate a nonlinear model and is of high accuracy. Moreover, it also has an extremely high fitness such that it is able to process different types of variable inputs. MLP also has its drawbacks associated with that since the hidden layers of MLP can be one or a multiple of layers, and it also requires the setting of its learning rate and parameters; therefore, it can be extremely time consuming.

#### 2.2.4. Rough Set Theory (RS)

In 1982, Pawlak proposed the rough set theory, which had been proved to be an effective mathematical tool for exploring data model [33]. It is a mathematical method for processing inaccurate, uncertain, and incomplete data. It uses the analysis and inference on data to discover the implied knowledge and to disclose potential patterns. Its core ability is to use the equivalence relation to divide a target set. It mainly uses the difference set of the lower approximations and upper approximations in the set theory, based on the concept of conditional probability, to perform calculation on the set already classified in order to obtain an objective result. The basic concept of rough set is to obtain the core set via reduction based on the discernible matrix established from the entire data system and to establish the result on the two-dimensional decision table of criteria attribute, research subject, and decision attribute [34]. Since the rough set theory proposed by Pawlak in 1982, it has provided an excellent research method for many researchers and has achieved rapid development. Applications related to the rough set theory in various fields include disease identification model [35], medication knowledge [36], stock market forecast [37], business decline prediction [38], customer relationship [39], and building windward surface value analysis [40].

#### 2.2.5. Radial Basis Function Network

Radial basis function network (RBF network) is an artificial neutral network using radical basis function as the activation function. The output of the radial basis function network is a linear combination of the inputted radial basis function and neural element parameters. The radial basis function network has numerous applications, including the function approximation, time-series prediction, classification, and system controls. Broomhead and Lowe [41] first established the radial basis function network; the radial basis function network typically includes three layers: input layer, hidden layer, and an output layer of a nonlinear activation function and linear radial basis neural network. The input can be modeled as a real vector, and the output is a scalar function of the input vector. The difference between the radial basis function network and the multilayer perception relies in that the function of the hidden node of the multilayer perception (MLP, including BP) uses a linear function, and the activation function uses a Sigmoid function or a step function. The most prominent characteristic of RBF network is that the hidden node basis function uses a distance function (such as Euclidean distance), and it uses the radial basis function (such as Gaussian function) as the activation function. In RBF network, the number of hidden layer neural elements is equivalent to the number of the radial basis function, which is also equivalent to the number of centers. Consequently, during the construction of radial basis artificial neural network, the number of centers of the radial basis function determines the size, complexity, and computation processing efficiency of the network, wherein the center location of the radial basis function can affect the convergence rate of the network.

#### 2.2.6. *K*-Nearest Neighbor (KNN)

KNN algorithm [42] is a relatively mature theoretical method, which is also one of the easiest machine learning algorithms and an algorithm that is most widely used today with high effectiveness and easy manipulation. The concept of KNN classification algorithm is a method for classifying nearest training samples in a characteristic space. In other words, it searches for *K* number of neighbors nearest to the new data having instances of the same class and high similarity among each other such that similarity of known classes of instances can be calculated in order to evaluate the possible classification of unknown classes of instances. Similarity is calculated by using distance functions, in which smaller distance refers to greater similarity. To understand the operation of KNN, we provide further explanations on the basic steps of the KNN algorithm as follows:

1. Determine parameter *k*, referring to the number of nearby neighbors.
2. Calculate the distance of the query-instance and all of the training samples.
3. Arrange the distances in step (2) in order, and determine the nearest neighbor based on the *k*th smallest distance.
4. Gather the class of the nearest neighbor.
5. Use the simple majority determination of the nearest neighbor class as the prediction value for query-instance.

### 2.3. Gene Expression Programming (GEP)

Gene expression programming (GEP) was proposed by Ferreira in 2001 [3]. Such method combines the advantages of the genetic algorithm (GA) and genetic programming (GP) such that it is a genotype/phenotype genetic algorithm. It is able to separate the evolutionary process and evaluation, encoding into a chromosome of a fixed length first, and then expressing in tree structure of different sizes and shapes at the time when the adaptive values are evaluated. Therefore, the method is of the simplicity of genetic algorithm and the function of the genetic programming at the same time such that, in terms of solving nonlinear problems, it has excellent data processing ability and exploration ability. In other words, GEP is one of the stable linear-GP techniques. GEP is mainly constructed by function set, terminal set, fitness function, control parameters, and termination criteria. Such algorithm utilizes text strings of fixed length and uses tree structures of different sizes and shapes to express the solution for the problem; this tree is known as the Express Tree. The characteristic of GEP relies in that it is able to construct a complicated evolution procedure into a multiple of subprocedures. Each GEP gene includes a list of any element symbols of fixed length, such as function set {+, −, *∗*, /, sin} or terminal set {*x*, *y*, *z*, −2}.

In the genetic algorithm [43, 44], the most important part is to express the problem applied in chromosomes, and the set of all of the solutions of the problem is called a population. In the population, each solution is a chromosome; therefore, a population can be seen as a set of chromosomes. Genes of a fixed number form each chromosome, and each gene represents a certain independent variable; therefore, each solution uses such variables of fixed numbers to express its characteristic. The gene expression programming combines advantages of the genetic algorithm and the genetic programming, which is identical to the genetic algorithm and genetic programming. At first, a set of initial population is generated randomly, and the quantity of the chromosomes in the population is set according to different problems. Next, the chromosomes are expressed in an Express Tree structure, and the adaptive value function set is used to calculate the adaptive value of each chromosome. During the calculation process of the adaptive function, greater adaptive value means that it fits our requirements on the solution of the problem better, and the contrary is false. Based on such standard, excellent chromosomes are preserved, and the chromosomes of poor qualities are eliminated, and the genetic operations of mutation, transformation, and reconfiguration are used to generate the next generation of new population. Repeat the aforementioned process and iteration until the termination criteria set is reached, and the computation is then stopped.

## 3. Proposed Method

Since 1966, there have been many methods used in predicting company bankruptcy and financial crisis, and many research results indicate that artificial intelligence yields better results than the traditional statistical method does. Despite that many statistical methods and artificial intelligence technologies have been used in establishing prediction models for financial crisis over the past years, only a few have used the time-series model. The data of financial crisis is seasonal time-series data, and financial data is nonlinear and unstable, and economic and financial systems are changed due to changes of model structures and behaviors. As a result, for different times and data, different time-series model would be required to interpret the evidence-based data. To allow the research data to be objective, this study used objective financial ratio as a research variable. In addition, to allow the class tag (financial healthy/crisis) of the research data to have balance class, this study redefined the term of financial distress based on the researches done by Chen et al. [45]. Finally, the research is based on the assumption that the profitability of a company does not vary differently in the four seasons. In other words, this study employed the asset profitability [46] to redefine the financial crisis as follows: assuming the quarterly asset profitability of a company is greater than zero, then we define that the company is healthy; whereas if its quarterly asset profitability is smaller than zero, then we define that the company is in financial distress. The asset profitability refers to the value obtained from the total income before tax divided by the total asset, which is a common method used for measuring the profitability of a company.

This paper utilized the nonlinear attribute selection to expedite the convergence of the gene expression programming. In addition, the reasons for establishing a financial crisis prediction model include the following: (a) high dimensional data cannot be handled with ease. For example, financial ratio includes a lot of attributes in a financial statement. To overcome this problem, this study proposed a new integrated attribute selection method to rank the ordering of attributes in order to reduce the data dimension and to establish a nonlinear prediction model and to determine whether the financial status of the company is healthy. (b) The three methods of gene expression programming (GEP), genetic algorithm (GA), and genetic programming (GP) have extremely similar evolution steps; moreover, GEP includes the properties of GA and GP, and GEP is able to achieve the objective of using simple encoding to solve complicated problems. The traditional GA uses the encoding method of a fixed length; consequently, it tends to yield poor results while dealing with complicated problems. GP uses a nonlinear and dynamic data structure for encoding such that it is able to handle complicated problems but, nevertheless, its operation is relatively complex. For GEP, it utilizes the chromosome encoding of a fixed length of GA and individuals of different sizes and shapes of GP in combination. In other words, the genetic part uses the method of GA for expression, and the tree structure of GP method is used for presenting the expression part. According to the article of “gene expression programming” by Ferreira in 2006, the evolution speed and effect of GEP have greater performance than those of GA and GP.

**Figure 1:**
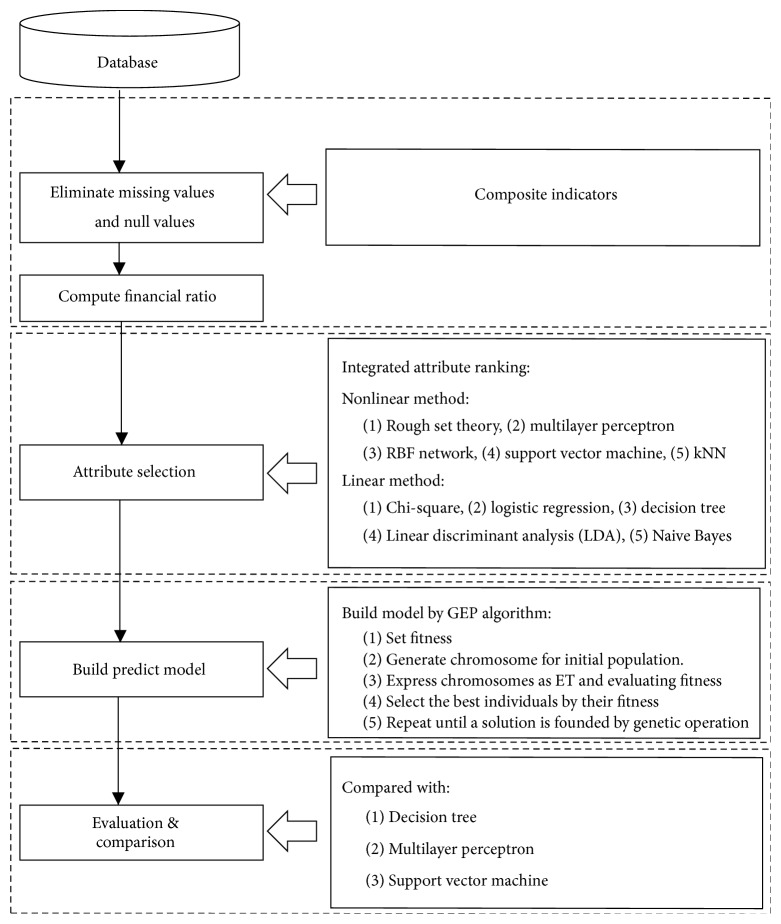
Flowchart of time-series financial crisis prediction model proposed.

The procedure of the research model proposed in this paper is shown in Figure 1. Firstly, the dataset of financial statements of corporate operation status is collected from Taiwan Economic Journal (TEJ), and the financial ratio is calculated from collected attribute data. Secondly, the ten attribute selection methods are used to select attributes, and the integrated attribute selection method proposed is used to rank the ordering of attribute. Thirdly, the gene expression programming is utilized to perform training, respectively, and the most optimal time-series financial crisis prediction model is established. Finally, the other prediction models are compared with the proposed model, and this study also compares the performance of the linear and nonlinear attribute selection methods.

*Computational Steps*. For understanding the proposed model easily, the followings split the proposed model into four steps to be introduced in detail.

**Step 1 (data preprocessing).**

For high dimensional data, preprocessing is an extremely important step to be performed. In geranial, the financial data and statements of companies are incomplete with missing values, interference values, or outliners. Therefore, this study practically collected the financial statements of the operation status of companies from the Taiwan Economic Journal, the related attribute data which possibly affects the financial crisis of the companies is obtained, and the collected dataset covers five-year period with 54 variables including a total of 13452 records. After the data is collected, the financial ratio is calculated and the redundant attributes, missing values, and empty values are deleted in this preprocessing step.

**Step 2 (attribute selection).**

The primary purpose of attribute selection is to select important and useful information from a vast amount of dataset and deleting unnecessary or interference attributes. Through attribute selection, the fewer attributes will be able to yield a more effective result. This step employed 10 most commonly used linear and nonlinear attribute selection methods, respectively, to select attributes, and the selected attributes in each attribute selection method are normalized by *W*_*i*_/max{*W*_*i*_} × 100 according to importance level to rank its ordering, wherein *w*_*i*_ refers to the importance level of the *i*th attribute under each attribute selection method. In this paper, the nonlinear attribute selection methods used include the nearest neighbor method, support vector machine, multilayer perception, radial basis function network (RBF network), and rough set theory. The linear methods used include the chi-square, logical regression, decision tree, linear discriminant analysis, and Native Bayes methods. To integrate the results of different attribute selection methods, this study proposed a new integrated attribute selection method to rank the ordering of attribute and provide a flexible selection method. The steps of the integrated attribute selection are introduced as follows:

  Step  1: using the financial healthy/distress of a company as a dependent variable (class label) and the other 34 attributes as independent variables, the attribute importance of each attribute under the optimized linear attribute selections and nonlinear attribute selection methods is calculated, respectively.
  Step  2: the above linear and nonlinear attribute selection methods are normalized by *W*_*i*_/max{*W*_*i*_} × 100 according to the importance level, wherein *w*_*i*_ refers to the importance level of the *i*th attribute under each attribute selection method. Next, the joins and disjoins under the linear and nonlinear attribute selection methods to select attributes, respectively. Here, the join is defined to be that when any two attribute selection methods have a common attribute, it would be a member of the join set. Accordingly, joins and disjoins are generated for the linear attribute selection methods. Similarly, joints and disjoins are also generated for the nonlinear attribute selection methods.

**Step 3 .**

**Figure 2:**
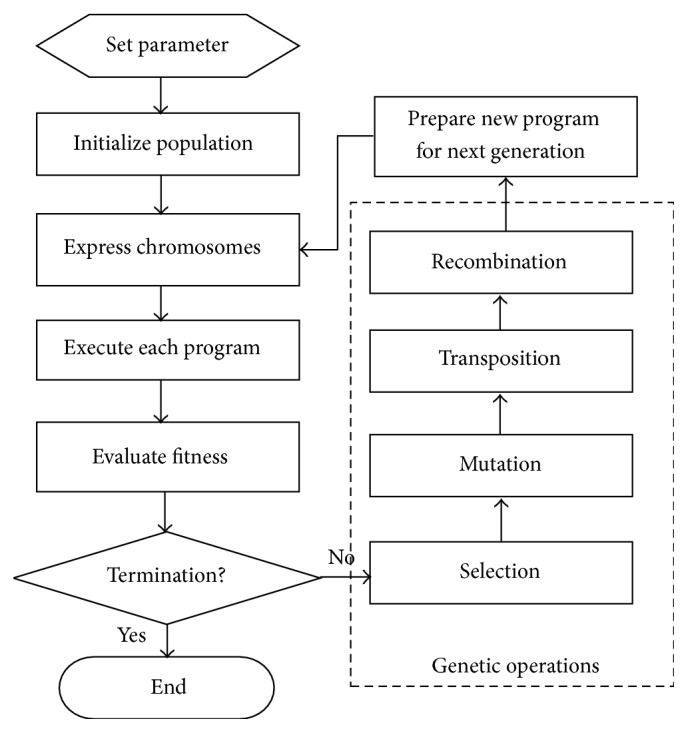
The procedure of computation step for gene expression programming.

To establish the optimal time-series financial crisis prediction model by GEP: according to the linear and nonlinear attribute selection methods as well as the integrated attribute selection methods, there are a total of 14 sets of attributes to build time-series financial crisis prediction model by using GEP algorithm, respectively, such that the optimal time-series financial crisis prediction model is established. The basic computation steps of the gene expression programming are listed as follows (the procedure of computation step as Figure 2):

(1) Set the fitness function for correct classification, and the fitness function of any GEP individual “*i*” is defined as
  (1) $$\begin{array}{c} {fitness}_{i}=\overset{N}{\underset{j=1}{\sum }}\left(\;c_{ij}=T_{j}\;\right), \end{array}$$
where *N* refers to the total number of the company in dataset, *T*_*j*_ refers to the target value for current financial status of company *j*, and *c*_*ij*_ refers to the financial status prediction of company *j* under GEP individual “*i*.”
(2) For the initial population, this step generates a chromosome of fixed length for each individual (candidate solution) randomly.
(3) Express the chromosome in a tree expression and evaluate the fitness of each individual.
(4) Perform reproduction and revision according to their fitness value, and select the most optimal individual.
(5) Based on a given number of generations, repeat the above Steps (2)–(4).

**Step 4 (evaluation and comparison).**

This study employed decision tree C4.5, MLP, and SVM data mining technologies to compare with the proposed model for classification performance. And based on objectiveness and standards, this study utilized three most common evaluation indices to measure the performance of financial crisis model: Type I error, Type II error, and accuracy.

## 4. Experiment and Comparison

To verify the proposed method, the financial statements of the company operation status published by Taiwan Economic Journal (TEJ) are collected, and related attribute data has been obtained. The data collection period covers the quarterly statements from 2008/1 to 2013/3, and there are total of 54 variables and 13,452 records. Through preprocessing by deleting redundant attributes, missing values, and empty values, there are a total 35 attributes (including class) with 8,278 records, among which 6,196 records are healthy companies and 2,535 records are companies with financial distress. In order to verify the performance, this study conducts the time-series experiment by sequential quarter. The total financial distress dataset covers 21 quarters for each company and the experimental dataset is partitioned into the former 14 quarters as training dataset and the last 7 quarters as testing dataset. The variables and the number of records for time-series dataset are shown in Table 3.

The term definition of the 35 attributes and financial ratio formula are shown in Table 4 [25]. Based on the proposed computational steps in Section 3, the results of 14 attribute selection methods are shown in Table 5. In evaluation, this study calculates the selected attributes of 14 attribute selection methods combined with six classifiers, respectively; then the results of three evaluation indices (Type I error, Type II error, and accuracy of the classification) are listed in Table 6. Based on Tables 5 and 6, the findings are explained as follows:

1. Attribute selection: from Table 5, the attributes *X*1, *X*4, and *X*30 are the higher frequency attributes, because the three attributes appear greater than 10 frequencies in 14 attribute selection methods. The three financial ratios are ROA(A)-EBI%, Return on Equity%-A, and Net Income%.
2. Accuracy: based on the selected attributes from 14 attribute selection methods combined with six classifiers, respectively, the results show that the accuracy of the GEP classifier is better than the other listing classifiers as Table 6. In terms of the attribute selection method, the decision tree attribute selection method yields better accuracy than the other methods. In addition, the proposed model utilizes decision tree attribute selection method combined with the GEP classifier that has the highest accuracy.
3. Type I error: from six different classifiers, MLP has the best performance in Type I error, and the performance of the RBF network attribute selection method is better than the other listing methods; therefore, the combination of the RBF network attribute selection method and MLP classifier has the optimal performance for Type I error.
4. Type II error: GEP classifier has the best performance in Type II error, and the logistic attribute selection method performs better than other methods in Type II error. Therefore, the combination of the logistic attribute selection method and GEP classifier has the optimal performance for Type II error.
5. Synthetic evaluation in three evaluation indices altogether: in different classifiers as Table 6, GEP is the best result, and linear attribute selection methods perform better than nonlinear attribute selection methods. It means that the proposed model in this research is advantageous. In addition, under the four attribute selection methods of joins and disjoins in linear and nonlinear attribute selection, the three evaluation indices are averaged, respectively, we can find that linear join attribute selection has better performance for accuracy and Type 1 error as Table 6.

At last for the advantages of GEP algorithm, the computational results can provide the tree structure and equation to user for easily understanding of financial distress prediction model. Therefore, this study also presents the two expressing formats in this section. After GEP computing, only five attributes, *X*1, *X*12, *X*22, *X*33, and *X*34, are employed, and the related coefficients for financial distress prediction model are

(2) G2C9=5.87152059389019,G4C5=−6.13330484939116,G4C8=9.18698690755943,G4C4=8.3135471663564,G4C9=9.03073213904233.

**Figure 3:**
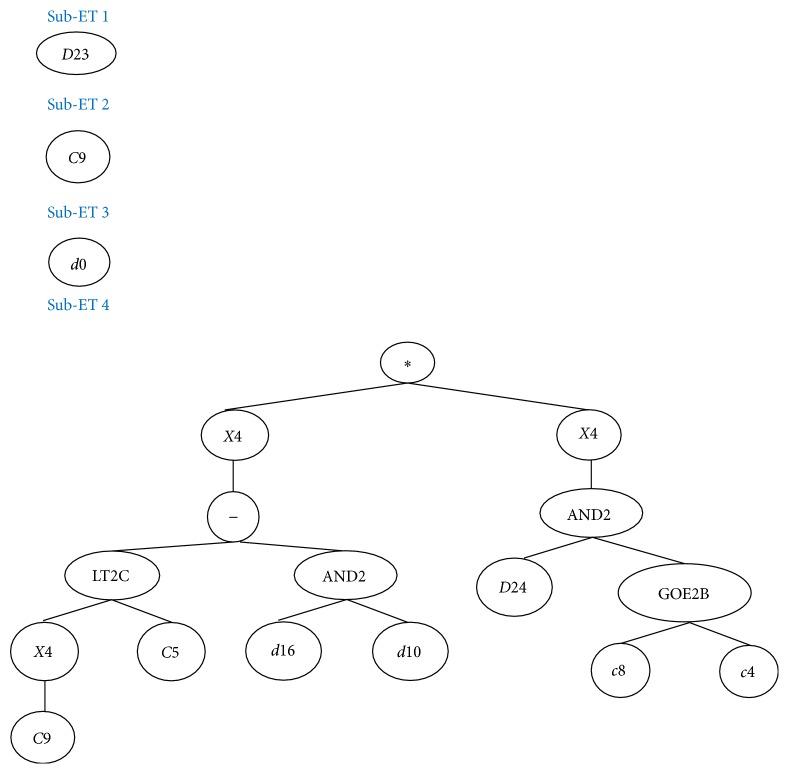
The tree structure of the proposed GEP model.

Then the tree structure of proposed model is shown as Figure 3, and the equation of financial distress prediction model is shown as

(3) $$\begin{array}{c} y=\left\{\begin{array}{c} 0,\\ d\left(x_{33}\right),\\ y+G2C9,\\ y+d\left(x_{1}\right),\\ y+LT2C\left({\left(G4C9\right)}^{4},G4C5\right)-AND2{\left(d\left(x_{22}\right),d\left(x_{12}\right)\right)}^{4}*AND2{\left(d\left(x_{34}\right),GE2B\left(G4C8,G4C4\right)\right)}^{4}, \end{array}\right. \end{array}$$

where *y* is GEPModel class. If  *y* ≥ *d* (*d* is rounding threshold); AND2(*x*, *y*) denotes that if ((*x* ≥ 0) and (*y* ≥ 0)), then AND2(*x*, *y*) = 1; LT2C(*x*, *y*) denotes that if (*x* < *y*), then (*x* + *y*); else (*x* − *y*); GE2B(*x*, *y*) means that if (*x* ≥ *y*), then GE2B(*x*, *y*) = 1; *d*(*x*_*i*_) represents *x*_*i*_ attribute value.

## 5. Conclusion

This study has presented the results of the bankruptcy prediction model in Section 4. To compare with the shortcoming of some of previous researches in the Introduction, we can find that this study has overcome some shortcomings: (1) this study adopted objective selected index, rather than subjective selected index, (2) the variables need not to follow relevant assumptions, (3) most of the past researches use the linear attribute selection method or no attribute selection to build prediction model; however, this research uses nonlinear attribute selection method, and (4) this research has proposed a novel seasonal time-series model based on gene expression programming for predicting the financial distress of companies. The experiment results indicate that the proposed model performs better than the other listing classifiers, and linear attribute selection methods perform better than nonlinear attribute selection methods. It means that the proposed method has relative advantages in predicting the financial distress of companies. Since financial statements are quarterly statements, the attribute data affecting the financial crisis of companies is nonlinear and unstable time-series data with fluctuations. The result indicates that the prediction outcome of the artificial intelligence GEP proposed in this research has relative stability for accuracy, Type I error, and Type II error.

## Figures and Tables

**Table 1:** Financial crisis related researches for research methods and results.

Author	Research methods	Data period	Sample ratio (crisis : health)	Accuracy
Frydman et al. [17]	Repeated segmentation logic	1971–1981	58 : 142	85.0–94.0

Mossman et al. [18]	LR	1970–1976	Moody's Industrial Manual 23 : 23	82.6–84.9

Atiya [19]	NN	1993–1996	US 444 : 716	60.0–90.8

Chen [20]	PCA + DT, PCA + LR	2000–2007	Taiwan 50 : 50	85.1–97.0

Li et al. [21]	RSBL, MDA, Logit, Probit	N/A	China 135 : 135,	71.6–88.5

Korol [2]	MDA, DT, NN	1993–1996	Warsaw 50 : 135 Latin America 60 : 30	74.1–96.8

Geng et al. [22]	DT, SVM, NN	Open dataset	China 344 : 344 Taiwan 220 : 220 Australian 307 : 382 German 700 : 300	70.9–92.1

Liang et al. [23]	SVM, RBF SVM, *k*-NN, NB, CART, MLP	Open dataset	China 344 : 344 Taiwan 220 : 220 Australian 307 : 382 German 700 : 300	70.9–92.1

MDA: multivariate discriminant analysis, DT: decision tree, NN: neural network, PCA: principal component analysis, LR: logistic regression, NB: Naive Bayes, MLP: multilayer perceptron neural network, CART: classification and regression tree, SVM: support vector machines, and RSBL: random subspace binary logit.

**Table 2:** Financial ratio used in related organization and researches.

Source	Financial ratio name
Financial holding company	(1) Operating capacity, (2) profitability, (3) financial structure, (4) solvency (5) Cash flow, (6) leverage, (7) capital adequacy

Stock exchange information observatory	(1) Financial analysis, (2) profit analysis, (3) gross profit ratio (4) inventory turnover ratio, (5) accounts receivable turnover

Major industries in Taiwan	(1) Financial structure, (2) solvency, (3) operational effectiveness, (4) profitability, (5) multiples, (6) asset-liability analysis, (7) cash flow analysis

Beaver [4]	(1) Cash flow ratio, (2) net profit ratio, (3) debt ratio, (4) current ratio, (5) quick ratio, (6) turnover ratio

Altman [10]	(1) Ratio of operating funds to total assets (2) Ratio of retained earnings to total assets (3) Ratio of net profit before tax to total assets (4) Ratio of equity market price to total liabilities (5) Ratio of net sales to total assets

Foster [7]	(1) Liquidity ratio, (2) capital structure ratio, (3) profitability ratio, (4) turnover rate

Wu [24]	(1) Financial structure, (2) solvency, (3) operational capacity, (4) profitability (5) Cash flow ratio (short-term solvency)

Li et al. [21]	30 financial ratios

Korol [2]	14 financial ratios

Geng et al. [22]	31 financial ratios

**Table 3:** Variables and records of time-series dataset.

Dataset	Variable	Record
Original data	54	13,452
Feature selection	35 (including class)	8,278
Training	35 (including class)	5,518
Testing	35 (including class)	2,760

**Table 4:** Definition and formulate of variable [25].

Financial ratios	Formulate	Variables
ROA (A) before tax interest	(Continued operating profit and loss + interest expense *∗* (1–17%))/total average assets *∗* 100 (%)	*X*1

ROA (B) before tax interest depreciation before	Pretax interest before depreciation recurring net profit/total assets *∗* 100 (%)	*X*2

ROA (C) pretax interest before depreciation	Pretax interest before depreciation recurring net profit/total assets *∗* 100 (%)	*X*3

ROE (A) after tax	Continue business unit profit and loss/average net *∗* 100	*X*4

Operating Expense Ratio	Operating expenses/net operating income *∗* 100 (%)	*X*5

Cash flow ratio	Cash flow/current Liabilities from operations *∗* 100 (%)	*X*6

Cash flow per share	(Cash flows from operations − dividends issued by special units)/weighted average number of shares	*X*7

Turnover per share (yuan)	Net operating income/(ordinary share capital + special share capital + share dividends to be distributed − treasury shares *∗* denomination) *∗* denomination	*X*8

Revenue growth rate	(Net operating income − net operating income for the same period of previous year)/ABS (net operating income for the same period of previous year) *∗* 100 (%)	*X*9

Pretax net profit growth	(Net profit before tax − net profit before tax for the same period of previous year)/ABS (net profit before tax for the same period of previous year) *∗* 100 (%)	*X*10

Total assets growth rate	(Total assets − total assets of the same period of last year)/ABS (total assets of same period of previous year) *∗* 100 (%)	*X*11

Current ratio	Current assets/current liabilities *∗* 100 (%)	*X*12

Quick ratio	(Cash and cash equivalents + financial assets at fair value through profit or loss − current + available-for-sale financial assets − current + held-to-maturity financial assets − current + hedged derivative financial assets − current + measured financial assets − floating + bond investments with no market-floating + accounts receivable & notes + other receivables + loans to others − mobility)/current liabilities *∗* 100 (%)	*X*13

Debt ratio%	Total liabilities/total assets *∗* 100 (%)	*X*14

Net worth/assets	Shareholders' equity/total assets *∗* 100 (%)	*X*15

The total number of assets turnover	Restore full year revenue/average total assets	*X*16

Accounts receivable turnover times	Restore full year revenue/average accounts receivable and bills	*X*17

Inventory turnover (times)	Operating costs/average inventory	*X*18

Fixed asset turnover times	Restore full year revenue/average real estate plant and equipment	*X*19

Working capital to total assets ratio	(Current assets − current liabilities)/total assets	*X*20

EBIT to total assets ratio	Pretax interest before net interest/total assets	*X*21

Cash flow to total liability ratio	Net cash flow-operating/total liabilities *∗* 100%	*X*22

Liquidity ratio	Current assets/total assets	*X*23

Cash/total assets	Cash/total assets	*X*24

Current liabilities/total assets	Current liabilities/total assets	*X*25

Fixed Assets/liabilities and shareholders' equity	Real estate plant and equipment/liabilities and shareholders' equity	*X*26

Shareholders' equity/total assets	Total shareholders' equity/total assets	*X*27

Total liabilities	Current liabilities + noncurrent liabilities	*X*28

Net profit after tax	Consolidated profit/loss/net income *∗* 100 (%)	*X*29

Pretax net profit margin	Net profit before tax/net operating income *∗* 100	*X*30

Operating margin	Operating margin/net operating income *∗* 100%	*X*31

Operating profit margin	Operating profit/net operating income *∗* 100	*X*32

ROE (B) - regular gain	(Continued operating unit profit or loss − gain on cheap purchases − disposal of property, plant, and equipment benefits + disposal of real estate, plant, and equipment losses − disposal of investment benefits + disposal of investment losses − gains or losses on financial assets (liabilities) measured at fair value through profit or loss + financial assets (liabilities) loss measured at fair value − asset evaluation benefit + asset evaluation loss − rotation interest deduction of financial assets + losses from impairment of financial assets − rotation interest of assets impairment + losses of assets impairment)/ABS (total shareholders' equity in the period + shareholders total equity) *∗* 2 *∗* 100 (%)	*X*33

EPS/total assets	EPS/total assets	*X*34

Health: P; distress: N	Asset earning power = earnings before taxes (EBT)/total assets	Class

**Table 5:** Selected attributes for 14 methods.

Linear							Nonlinear						
Chi-sq.	DT	KNN	LDA	Logit	Join	Disjoin	Nave	SVM	RBF	RS	MLP	Join	Disjoin
**X**1	**X**1	**X**1	**X**1	**X**1	**X**1	**X**1	**X**1			**X**1	**X**1	**X**1	**X**1
*X*2					*X*2	*X*2	*X*2			*X*2			*X*2
*X*3					*X*3	*X*3	*X*3			*X*3			*X*3
**X**4		**X**4	**X**4	**X**4	**X**4	**X**4			**X**4	**X**4		**X**4	**X**4
*X*5						*X*5				*X*5			*X*5
*X*7					*X*7	*X*7	*X*7			*X*7			*X*7
*X*8					*X*8	*X*8	*X*8			*X*8			*X*8
*X*9			*X*9		*X*9	*X*9				*X*9	*X*9		*X*9
*X*10	*X*10				*X*10	*X*10				*X*10			*X*10
*X*11	*X*11				*X*11	*X*11				*X*11			*X*11
*X*12						*X*12							
*X*13	*X*13				*X*13	*X*13				*X*13			*X*13
*X*16						*X*16							
*X*19	*X*19		*X*19		*X*19	*X*19				*X*19			*X*19
*X*20						*X*20							
*X*21						*X*21							
*X*22						*X*22				*X*22			
*X*23					*X*23	*X*23	*X*23	*X*23					*X*23
*X*24	*X*24				*X*24	*X*24	*X*24			*X*24			*X*24
*X*29	*X*29		*X*29		*X*29	*X*29			*X*29				*X*29
**X**30	**X**30	**X**30	**X**30		**X**30	**X**30		**X**30	**X**30	**X**30		**X**30	**X**30
*X*31						*X*31				*X*31			*X*31
*X*32		*X*32	*X*32		*X*32	*X*32				*X*32		*X*32	*X*32
*X*33						*X*33				*X*33			*X*33
*X*34		*X*34	*X*34		*X*34	*X*34	Nave			*X*34		*X*34	*X*34

**Table 6:** The results for financial distress dataset.

Feature selection	Classifier	Accuracy	Type I	Type II
Linear				
Chi-square	GEP	98.96	0.010916	0.009292
	Decision tree	98.85	0.01279	0.008168
	MLP	95.35	0.043108	0.054842
	SVM	97.94	0.009018	0.051282
	RBF	77.43	0.727498	0.018755
	KNN	87.57	0.225761	0.078842
Decision tree	GEP	99.06^*∗∗*^	0.009967	0.00813
	Decision tree	98.98	0.011369	0.007001
	MLP	97.43	0.024633	0.028005
	SVM	97.91	0.008543	0.051103
	RBF	75.21	0.838425	0.010161
	KNN	89.61	0.218475	0.057228
KNN	GEP	98.96	0.011391	0.00813
	Decision tree	98.95	0.011369	0.008168
	MLP	97.16	0.002369	0.092182
	SVM	95.18	0.060161	0.018673
	RBF	82.42	0.598425	0.048435
	KNN	98.14	0.025846	0.085834
LDA	GEP	98.96	0.011391	0.00813
	Decision tree	98.95	0.011369	0.008168
	MLP	96.52	0.001895	0.115519
	SVM	98.48	0.012228	0.032914
	RBF	85.14	0.493883	0.012481
	KNN	97.75	0.039478	0.013478
Logistic	GEP	99.02	0.011391	0.005807^*∗∗*^
	Decision tree	98.75	0.012912	0.011453
	MLP	97.60	0.002842	0.075846
	SVM	97.97	0.018475	0.024504
	RBF	95.17	0.147651	0.012779
	KNN	98.03	0.038432	0.013497

Nonlinear				
MLP	GEP	98.25	0.041766	0.034843
	Decision tree	97.87	0.006158	0.058343
	MLP	98.04	0.007106	0.050175
	SVM	94.64	0.009000	0.163361
	RBF	97.49	0.058742	0.012411
	KNN	97.09	0.052413	0.018754
Naive Bayes	GEP	98.52	0.010916	0.024390
	Decision tree	97.97	0.006158	0.054842
	MLP	95.78	0.040739	0.045508
	SVM	94.81	0.005685	0.165694
	RBF	88.58	0.283871	0.052137
	KNN	86.37	0.262475	0.092571
RBF network	GEP	98.99	0.00813	0.010916
	Decision tree	98.95	0.011369	0.008168
	MLP	91.00	0.000474^*∗∗*^	0.310385
	SVM	96.36	0.039792	0.028005
	RBF	97.70	0.048742	0.012378
	KNN	97.97	0.028773	0.012475
Rough set	GEP	99.02	0.010916	0.006969
	Decision tree	98.88	0.01279	0.007001
	MLP	93.93	0.074372	0.026838
	SVM	97.91	0.008543	0.051103
	RBF	78.07	0.728475	0.013418
	KNN	88.48	0.217582	0.073591
SVM	GEP	98.92	0.010067	0.012941
	Decision tree	98.85	0.011369	0.011669
	MLP	73.28	0.03837	0.169195
	SVM	98.11	0.016106	0.025671
	RBF	73.10	0.884217	0.023458
	KNN	69.13	0.592195	0.201728

Linear				
Join	GEP	98.96	0.010916	0.009292
	Decision tree	98.95	0.011369	0.008168
	MLP	97.20	0.002369	0.091015
	SVM	96.09	0.044529	0.025671
	RBF	78.09	0.724258	0.022889
	KNN	86.32	0.262479	0.091348
	Average	92.60^*∗*^	0.1759866^*∗*^	0.041397
Disjoin	GEP	98.99	0.010916	0.00813
	Decision tree	98.88	0.01279	0.007001
	MLP	92.38	0.085741	0.052509
	SVM	98.15	0.008068	0.027944
	RBF	77.43	0.732561	0.023457
	KNN	87.57	0.262877	0.083271
	Average	92.23	0.185492	0.033718^*∗*^

Nonlinear				
Join	GEP	98.96	0.011391	0.00813
	Decision tree	98.95	0.011369	0.008168
	MLP	97.16	0.002369	0.092182
	SVM	95.18	0.060161	0.01867
	RBF	82.42	0.60247	0.002431
	KNN	98.14	0.034237	0.012798
	Average	95.14	0.120332	0.023723
Disjoin	GEP	98.96	0.010916	0.009292
	Decision tree	98.85	0.012808	0.008168
	MLP	95.30	0.043108	0.054842
	SVM	97.94	0.009018	0.04878
	RBF	77.11	0.763741	0.012885
	KNN	86.94	0.242298	0.083179
	Average	92.51	0.180314	0.036191

*Note*. *∗∗* denotes the best result in accuracy, Type I, and Type II, respectively. *∗* denotes the better result for average of join and disjoin in accuracy, Type I, and Type II, respectively.
